# Expression of Concern: Antitumor Activity of Sorafenib in Human Cancer Cell Lines with Acquired Resistance to EGFR and VEGFR Tyrosine Kinase Inhibitors

**DOI:** 10.1371/journal.pone.0215109

**Published:** 2019-04-11

**Authors:** The PLOS ONE Editors

After publication of this article [1], the following concerns were raised about results reported in Figures 1, 3, 5, and 6:

- In Figure 1, lanes 2, 3, 4 of the CALU-3 MAPK blot appear highly similar to lanes 4, 3, 2 of the HCT-116 p-MAPK blot, with 180-degree rotation.
- In Figure 3, similarities were noted between the p-MAPK44/42 and pMEK blots for HCT-116 cells.
- In Figure 3, similarities were noted between the p445-BRAF and pMEK blots for CALU-3 cells.
- Duplicate results were presented in Figures 5 and 6 in error.

The original underlying data supporting results in this article are no longer available. The authors replicated the experiments for which concerns were raised using the same methods and conditions as in the original study [1]. Here, they provide updated versions of Figures 1, 3, 5, and 6 that report the replication experiments results, along with underlying data, as Supporting Information files. The authors noted that the replication experiments for Figures 5, 6 were approved by the Second University of Naples Animal Care and Use Committee under approval number IT2017/12187.

The *PLOS ONE* Editors issue this Expression of Concern to notify readers of the issues raised for the published figures and the unavailability of original data for this study.

## Materials and methods for Figure 1 and 3 replication experiments

Protein lysates, derived from cells following treatment, were obtained by homogenization in RIPA lyses buffer [0.1% sodium dodecylsulfate (SDS), 0,5% deoxycholate, 1% Nonidet, 100 mmol/L NaCl, 10 mmol/L Tris–HCl (pH 7.4), 0.5 mmol/L dithiotritol, and 0.5% phenylmethyl sulfonyl fluoride, protease inhibitor cocktail (Hoffmann-La Roche)] and clarification by centrifugation at 14,000 rpm for 15 min at +4°C. Protein samples containing comparable amounts of proteins, estimated by a modified Bradford assay (Bio-Rad), were subjected to Western blot and immunocomplexes were detected with the enhanced chemiluminescence kit ECL plus, by Thermo Fisher Scientific (Rockford, IL). Membrane pictures were taken by a ChemiDoc System (Bio-Rad, Hercules CA) and bands densities were analyzed with Image Lab Software (Bio-Rad). Equal aliquots of the same sample preparation were loaded and different antibodies were used to reprobe the same blot.

* The same antibodies were used in the published article and in the replication experiments. For tubulin mouse monoclonal anti- alpha-tubulin antibody was used (Sigma Aldrich Chemical, Saint Louis, Missouri, United States)

## Supporting information

S1 File Updated Figure 1. Updated Figure 1 and supporting raw blot images from replication experiments: Western blotting analysis of parental CALU-3 and HCT-116 cells (WT) and their TKI-resistant derivatives (ERL-R, GEF-R, VAN-R). Actin and tubulin were included as loading controls. The MAPK44/42, p-MAPK44/42, and Tubulin panels for both cell lines report data from replication experiments. Other results in the updated figure are the same as those reported in the original published figure [1]. For replication experiments, the control data (tubulin) were generated by reprobing the same membranes used for the corresponding total protein and phospho-protein blots. For the original experiments, the β-actin blots were conducted on separate membranes from the experimental blots using equal aliquots of the same sample preparations.
(PPTX)

S2 File Updated Figure 3. Updated Figure 3 and supporting raw blot images from replication experiments. The following panels report data from replication experiments: BRAF, p445-BRAF, Tubulin, MEK, pMEK, Tubulin for CALU-3 cells; MAPK44/42, p-MAPK44/2, Tubulin, MEK, pMEK, Tubulin for HCT-116 cells. Other results in the updated figure are the same as those reported in the original published figure [1]. For replication experiments, the control data (tubulin) were generated by reprobing the same membranes used for the corresponding total protein and phospho-protein blots. For the original experiments, the β-actin blots were conducted on separate membranes from the experimental blots using equal aliquots of the same sample preparations.
(PPTX)

S3 File Updated Figure 5 and 6. Updated versions of Figures 5 and 6. Updated figures are the results of a replication study conducted with the same methodology of the original experiments.
(PPTX)

S4 File Raw data. Raw data from in vivo replication experiments reported in Figures 5 and 6.
(XLS)
