# Supplementary material for: Expression of Concern: Antitumor Activity of Sorafenib in Human Cancer Cell Lines with Acquired Resistance to EGFR and VEGFR Tyrosine Kinase Inhibitors
Source: PLoS One. 2019 Apr 11;14(4):e0215109. doi: 10.1371/journal.pone.0215109 (PMC6459487; doi:10.1371/journal.pone.0215109)
Supplement: S1 File — Updated Figure 1 and supporting raw blot images from replication experiments: Western blotting analysis of parental CALU-3 and HCT-116 cells (WT) and their TKI-resistant derivatives (ERL-R, GEF-R, VAN-R). Actin and tubulin were included as loading controls. The MAPK44/42, p-MAPK44/42, and Tubulin panels for both cell lines report data from replication experiments. Other results in the updated figure are the same as those reported in the original published figure [1]. For replication experiments, the control data (tubulin) were generated by reprobing the same membranes used for the corresponding total protein and phospho-protein blots. For the original experiments, the β-actin blots were conducted on separate membranes from the experimental blots using equal aliquots of the same sample preparations. (PPTX) [file pone.0215109.s001.pptx]

## Slide 1
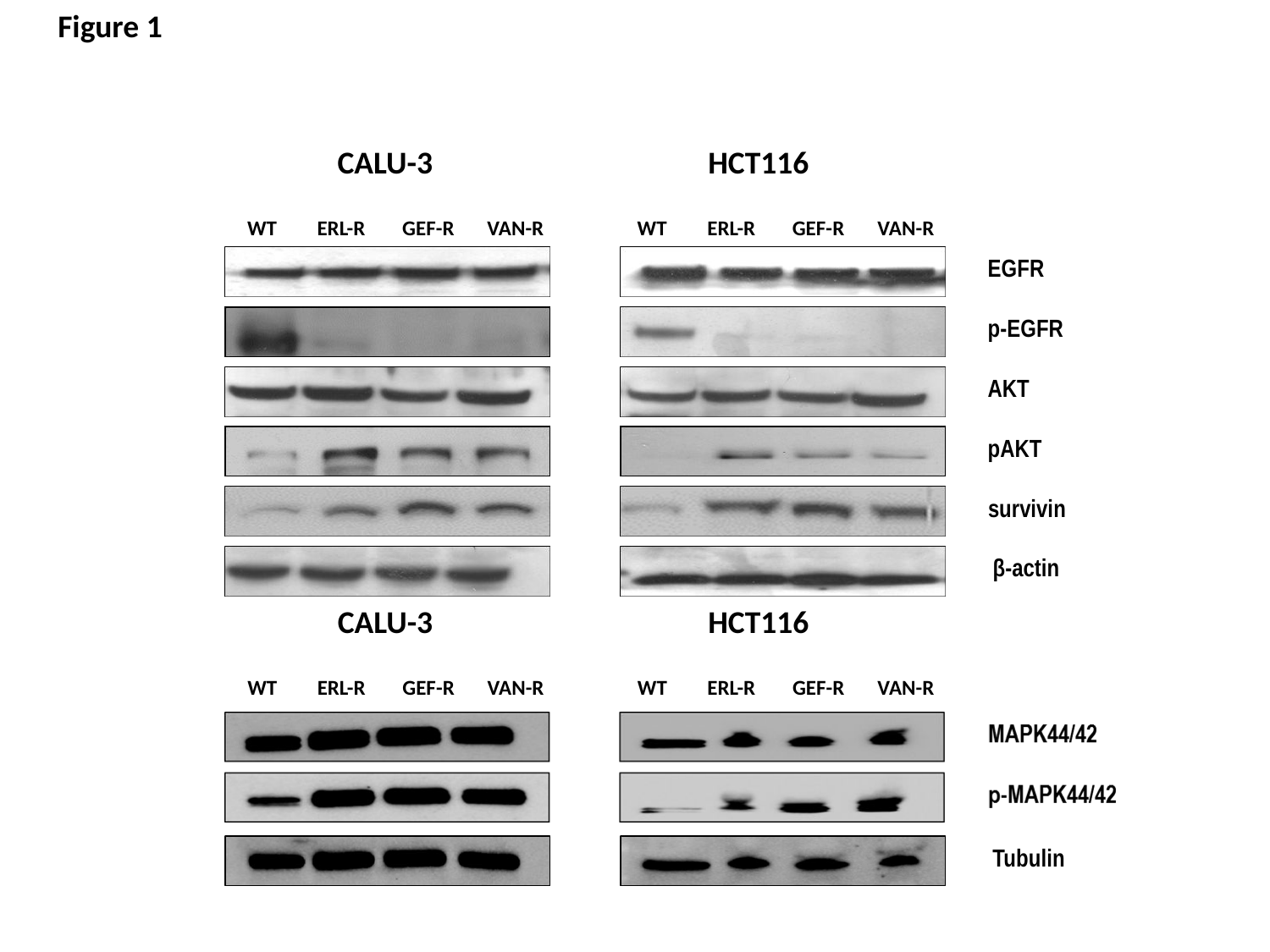

Figure 1
CALU-3
HCT116
WT
ERL-R
GEF-R
VAN-R
WT
ERL-R
GEF-R
VAN-R
EGFR
p-EGFR
AKT
pAKT
survivin
β-actin
CALU-3
HCT116
WT
ERL-R
GEF-R
VAN-R
WT
ERL-R
GEF-R
VAN-R
Tubulin

## Slide 2
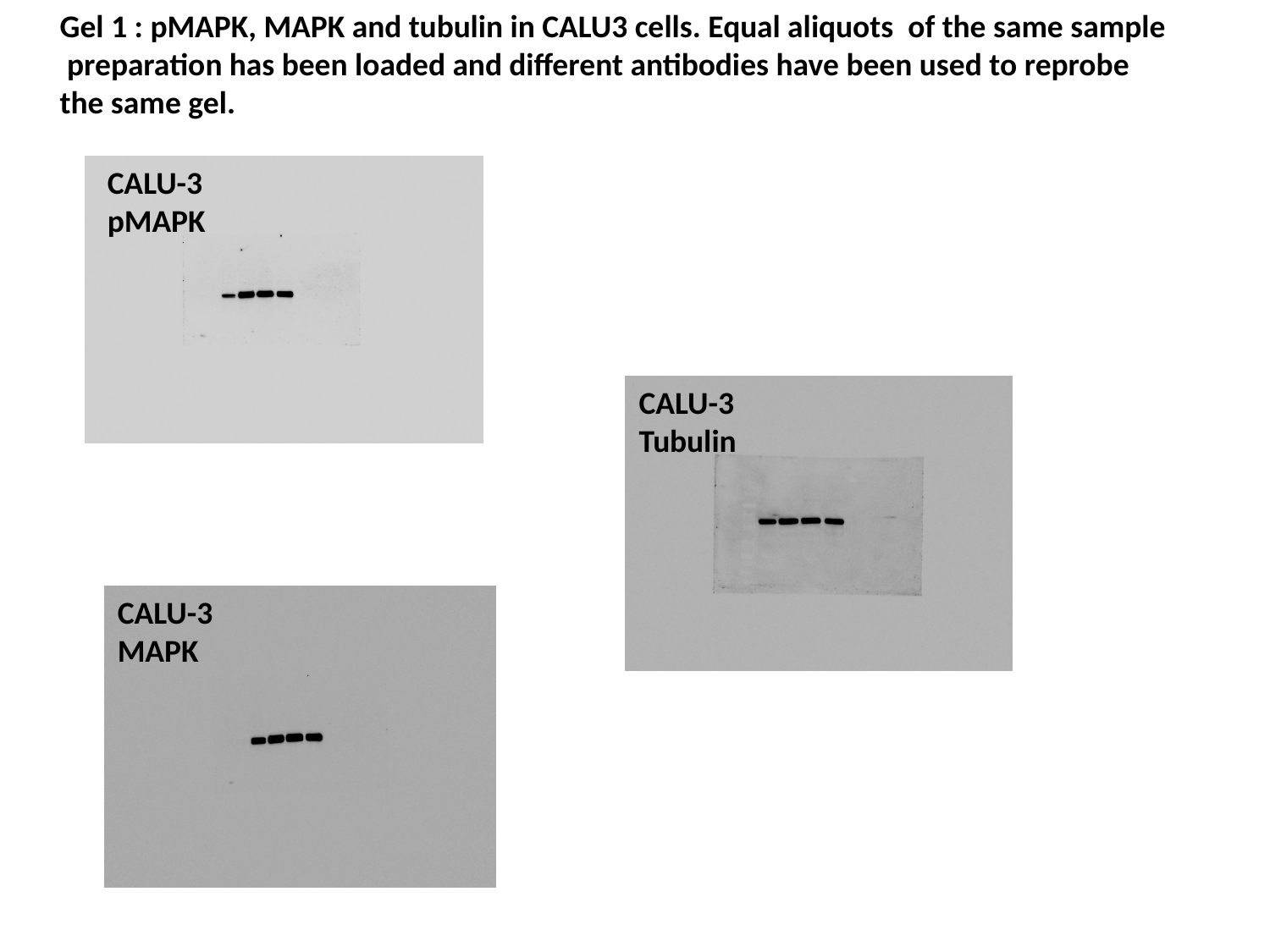

Gel 1 : pMAPK, MAPK and tubulin in CALU3 cells. Equal aliquots of the same sample
 preparation has been loaded and different antibodies have been used to reprobe
the same gel.
CALU-3
pMAPK
CALU-3
Tubulin
CALU-3
MAPK

## Slide 3
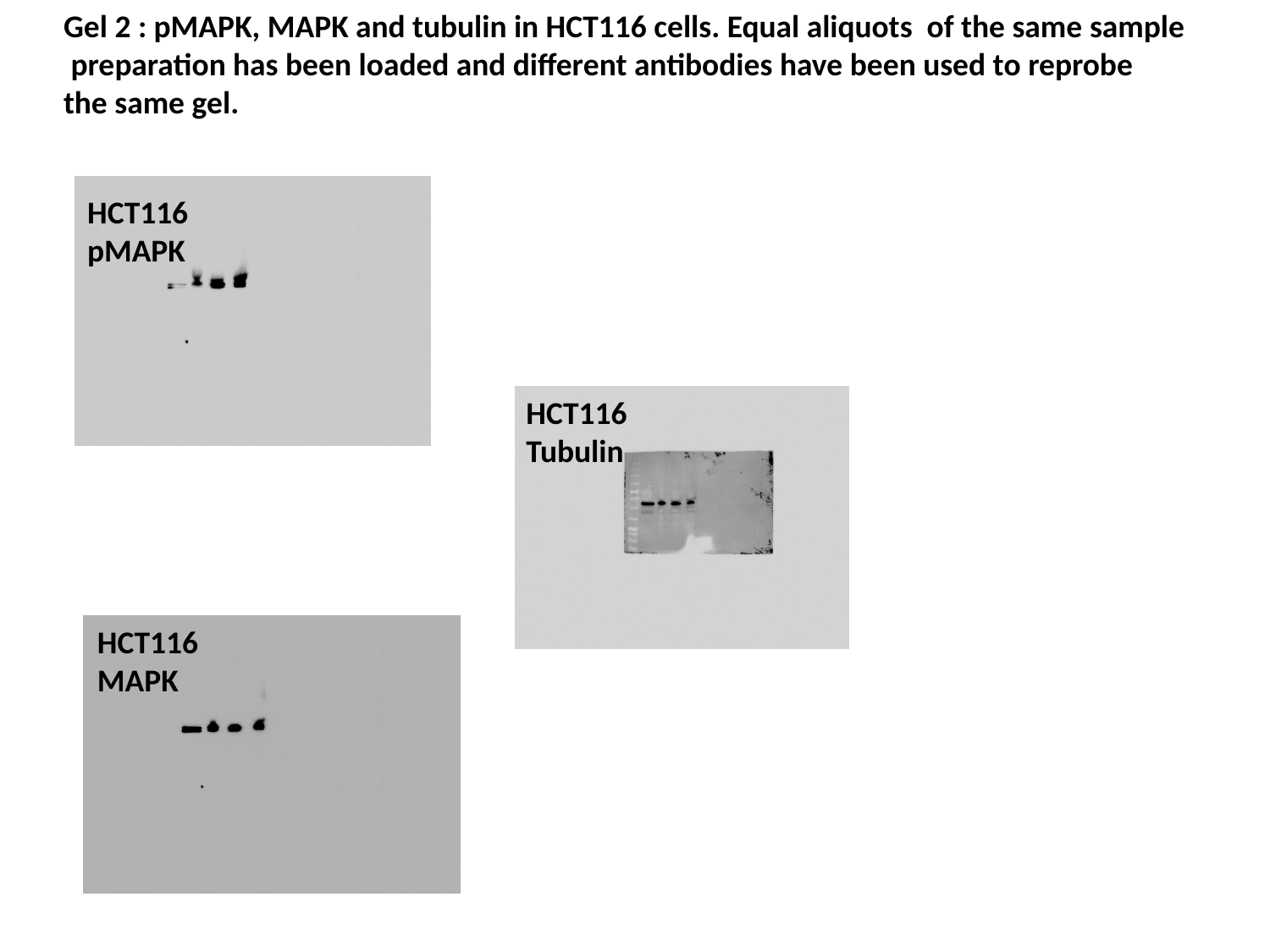

Gel 2 : pMAPK, MAPK and tubulin in HCT116 cells. Equal aliquots of the same sample
 preparation has been loaded and different antibodies have been used to reprobe
the same gel.
HCT116
pMAPK
HCT116
Tubulin
HCT116
MAPK
