# Supplementary material for: Expression of Concern: Antitumor Activity of Sorafenib in Human Cancer Cell Lines with Acquired Resistance to EGFR and VEGFR Tyrosine Kinase Inhibitors
Source: PLoS One. 2019 Apr 11;14(4):e0215109. doi: 10.1371/journal.pone.0215109 (PMC6459487; doi:10.1371/journal.pone.0215109)
Supplement: S2 File — Updated Figure 3 and supporting raw blot images from replication experiments. The following panels report data from replication experiments: BRAF, p445-BRAF, Tubulin, MEK, pMEK, Tubulin for CALU-3 cells; MAPK44/42, p-MAPK44/2, Tubulin, MEK, pMEK, Tubulin for HCT-116 cells. Other results in the updated figure are the same as those reported in the original published figure [1]. For replication experiments, the control data (tubulin) were generated by reprobing the same membranes used for the corresponding total protein and phospho-protein blots. For the original experiments, the β-actin blots were conducted on separate membranes from the experimental blots using equal aliquots of the same sample preparations. (PPTX) [file pone.0215109.s002.pptx]

## Slide 1
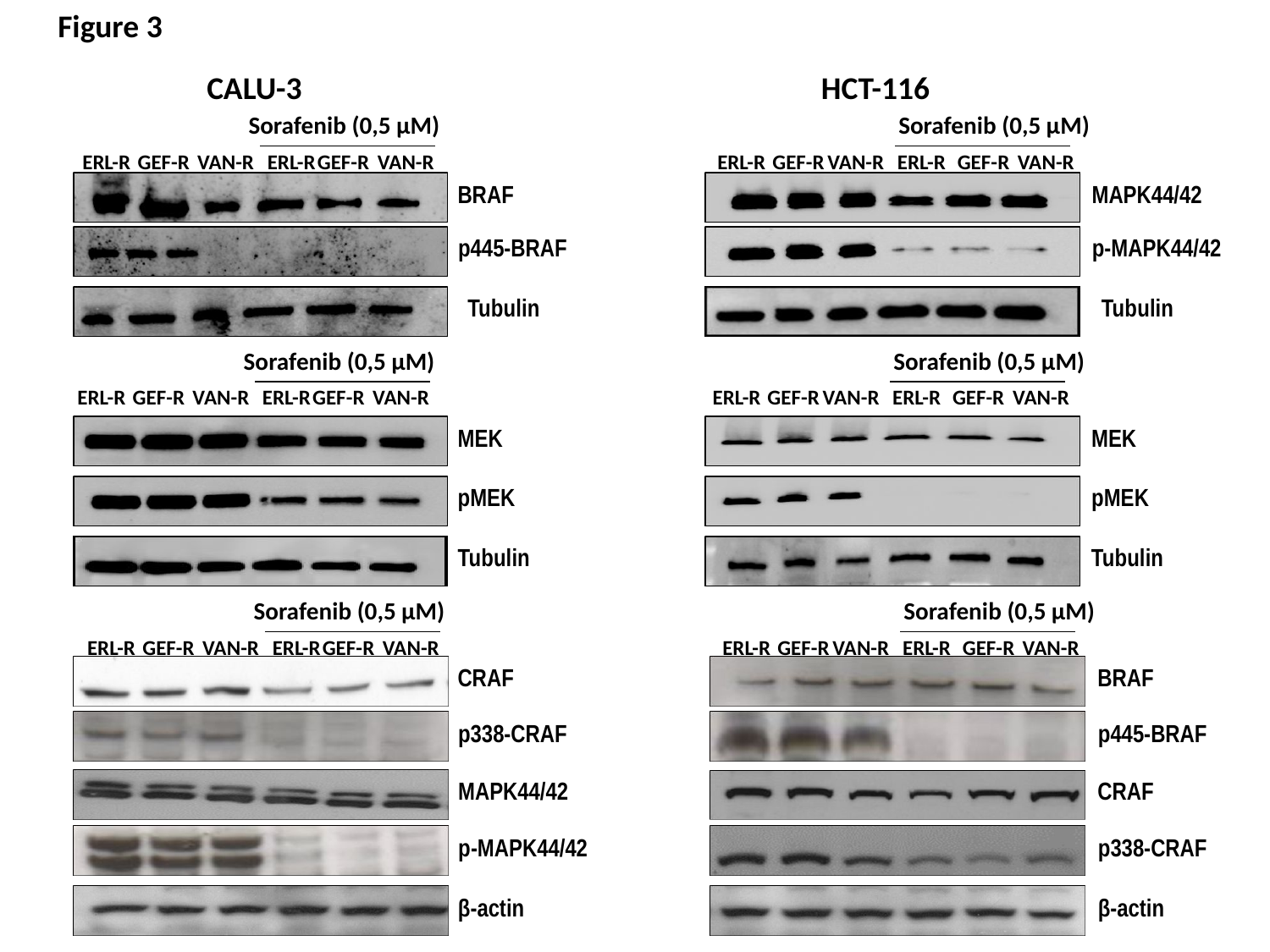

Figure 3
CALU-3
HCT-116
Sorafenib (0,5 µM)
Sorafenib (0,5 µM)
ERL-R
GEF-R
VAN-R
ERL-R
GEF-R
VAN-R
ERL-R
GEF-R
VAN-R
ERL-R
GEF-R
VAN-R
BRAF
MAPK44/42
p445-BRAF
p-MAPK44/42
Tubulin
Tubulin
Sorafenib (0,5 µM)
Sorafenib (0,5 µM)
ERL-R
GEF-R
VAN-R
ERL-R
GEF-R
VAN-R
ERL-R
GEF-R
VAN-R
ERL-R
GEF-R
VAN-R
MEK
MEK
pMEK
pMEK
Tubulin
Tubulin
Sorafenib (0,5 µM)
Sorafenib (0,5 µM)
ERL-R
GEF-R
VAN-R
ERL-R
GEF-R
VAN-R
ERL-R
GEF-R
VAN-R
ERL-R
GEF-R
VAN-R
CRAF
BRAF
p338-CRAF
p445-BRAF
MAPK44/42
CRAF
p-MAPK44/42
p338-CRAF
β-actin
β-actin

## Slide 2
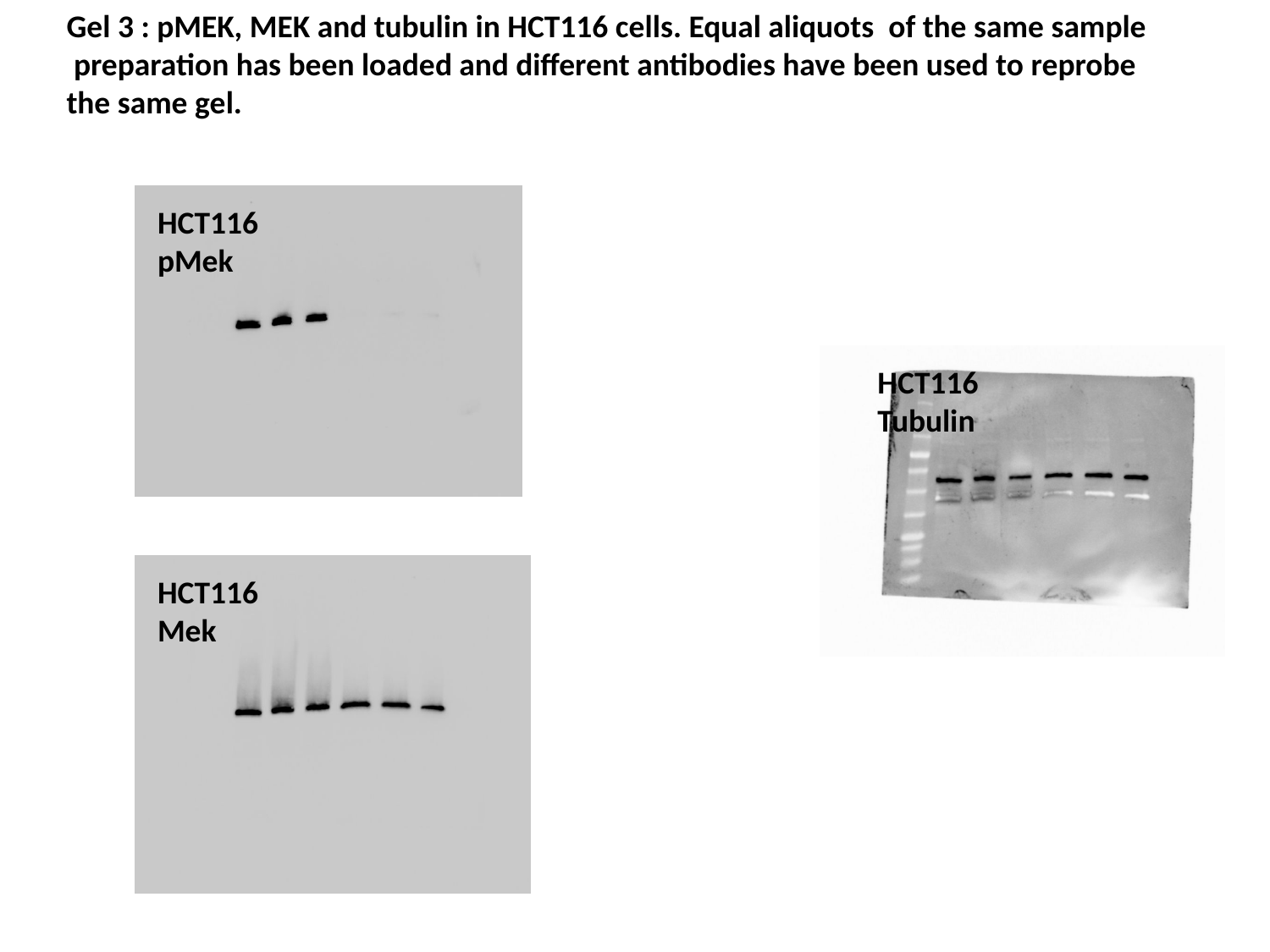

Gel 3 : pMEK, MEK and tubulin in HCT116 cells. Equal aliquots of the same sample
 preparation has been loaded and different antibodies have been used to reprobe
the same gel.
HCT116
pMek
HCT116
Tubulin
HCT116
Mek

## Slide 3
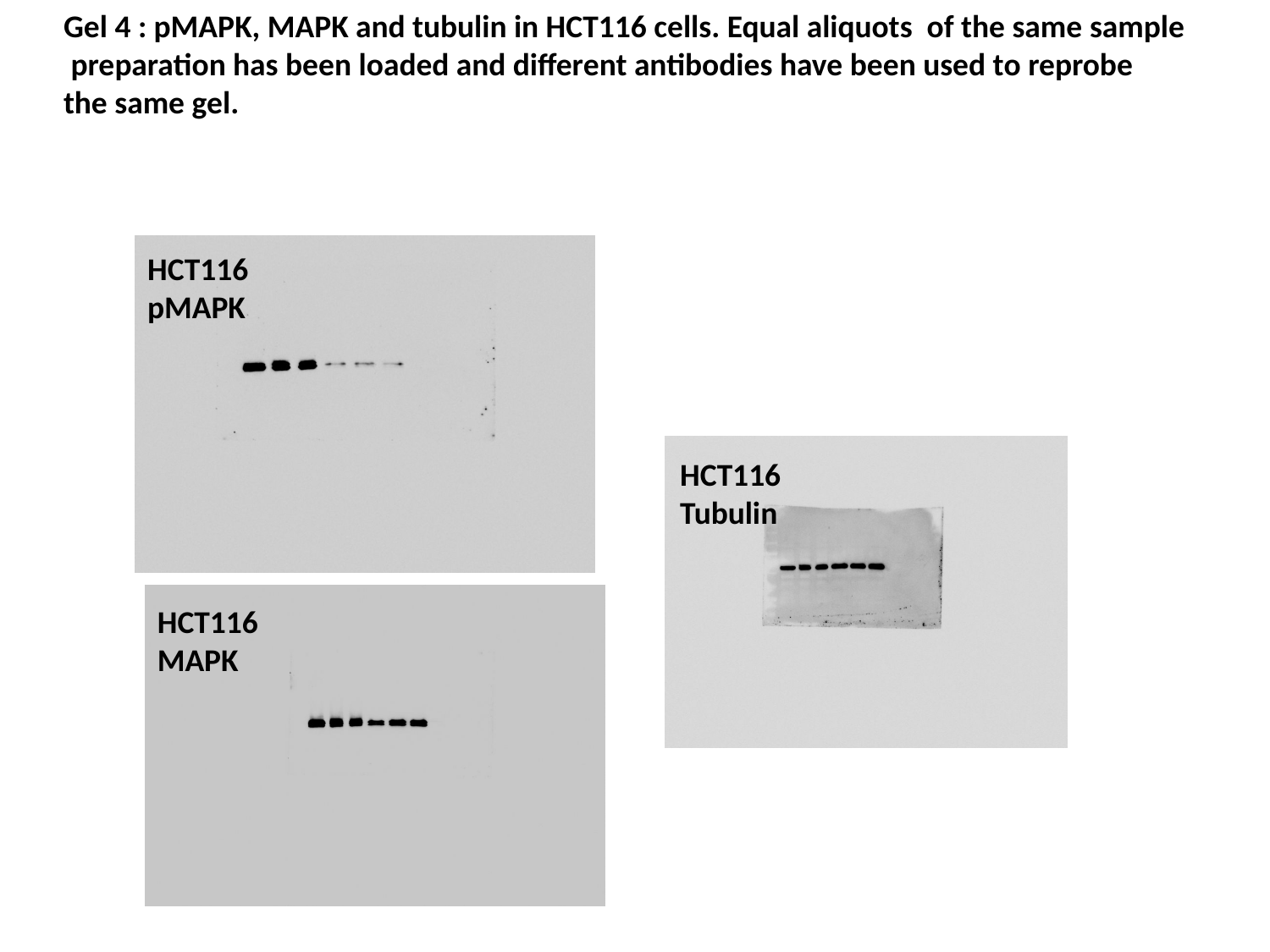

Gel 4 : pMAPK, MAPK and tubulin in HCT116 cells. Equal aliquots of the same sample
 preparation has been loaded and different antibodies have been used to reprobe
the same gel.
HCT116
pMAPK
HCT116
Tubulin
HCT116
MAPK

## Slide 4
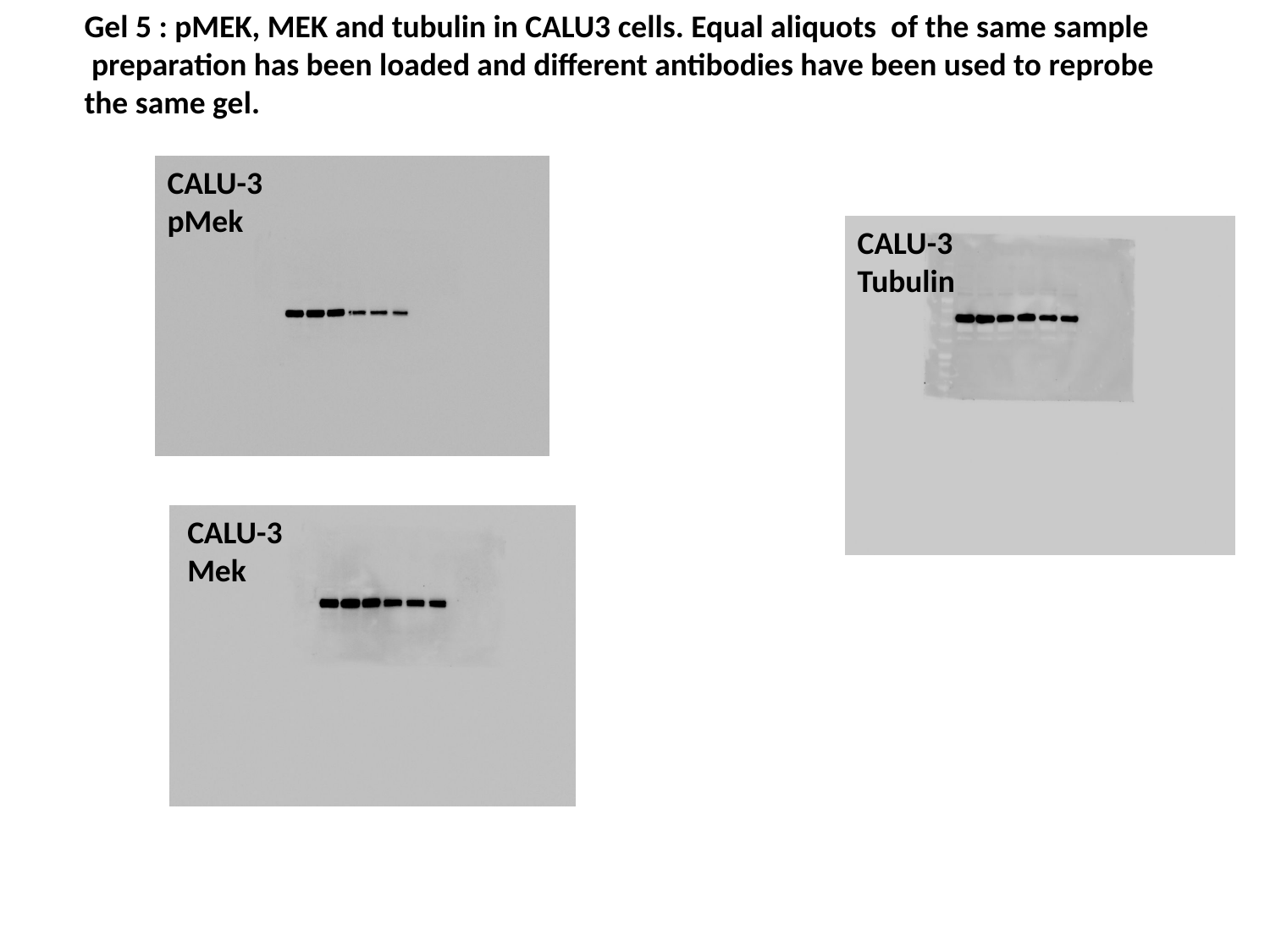

Gel 5 : pMEK, MEK and tubulin in CALU3 cells. Equal aliquots of the same sample
 preparation has been loaded and different antibodies have been used to reprobe
the same gel.
CALU-3
pMek
CALU-3
Tubulin
CALU-3
Mek

## Slide 5
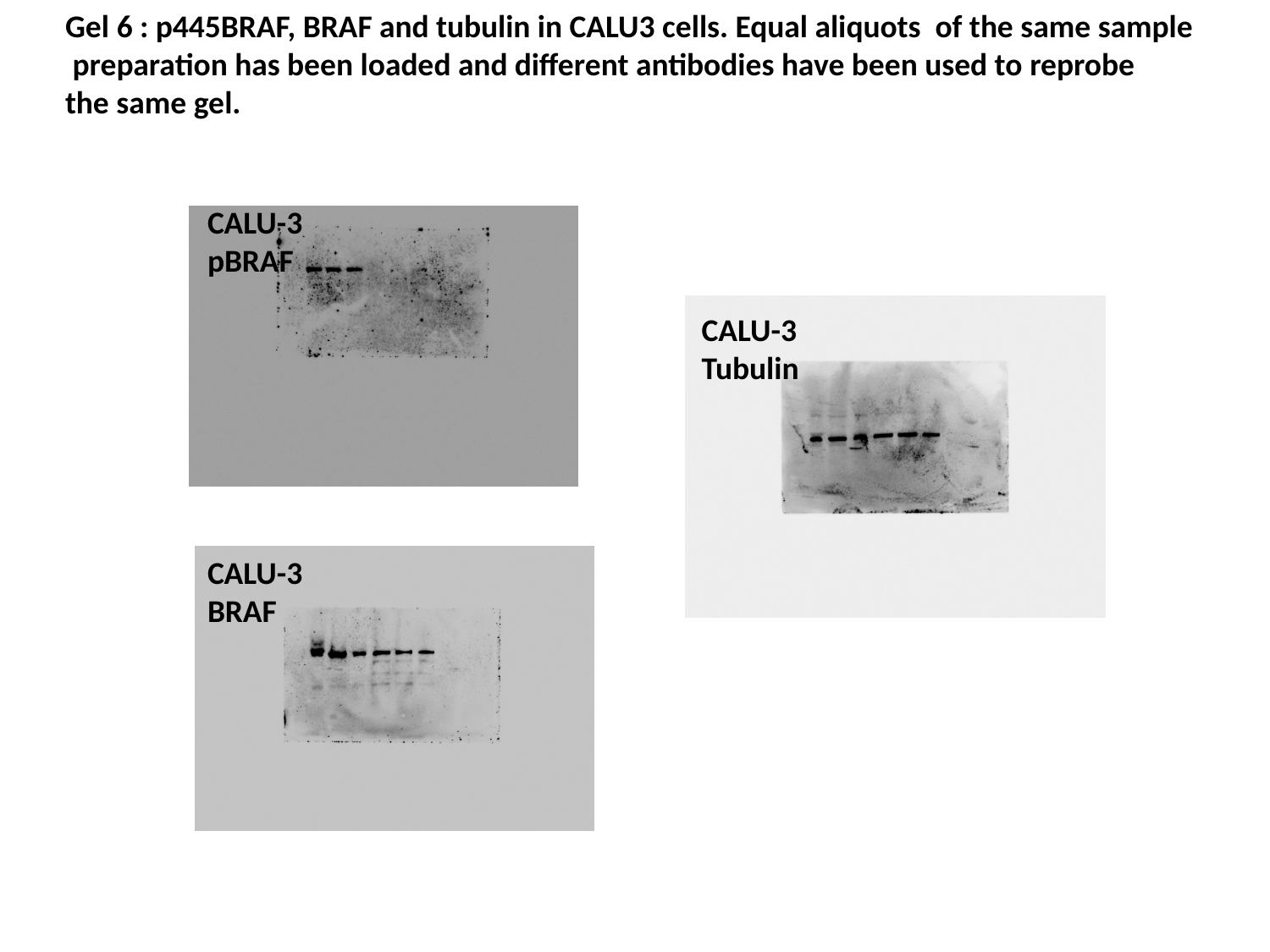

Gel 6 : p445BRAF, BRAF and tubulin in CALU3 cells. Equal aliquots of the same sample
 preparation has been loaded and different antibodies have been used to reprobe
the same gel.
CALU-3
pBRAF
CALU-3
Tubulin
CALU-3
BRAF
