# Supplementary material for: Expression of Concern: Antitumor Activity of Sorafenib in Human Cancer Cell Lines with Acquired Resistance to EGFR and VEGFR Tyrosine Kinase Inhibitors
Source: PLoS One. 2019 Apr 11;14(4):e0215109. doi: 10.1371/journal.pone.0215109 (PMC6459487; doi:10.1371/journal.pone.0215109)
Supplement: S3 File — Updated versions of Figures 5 and 6. Updated figures are the results of a replication study conducted with the same methodology of the original experiments. (PPTX) [file pone.0215109.s003.pptx]

## Slide 1
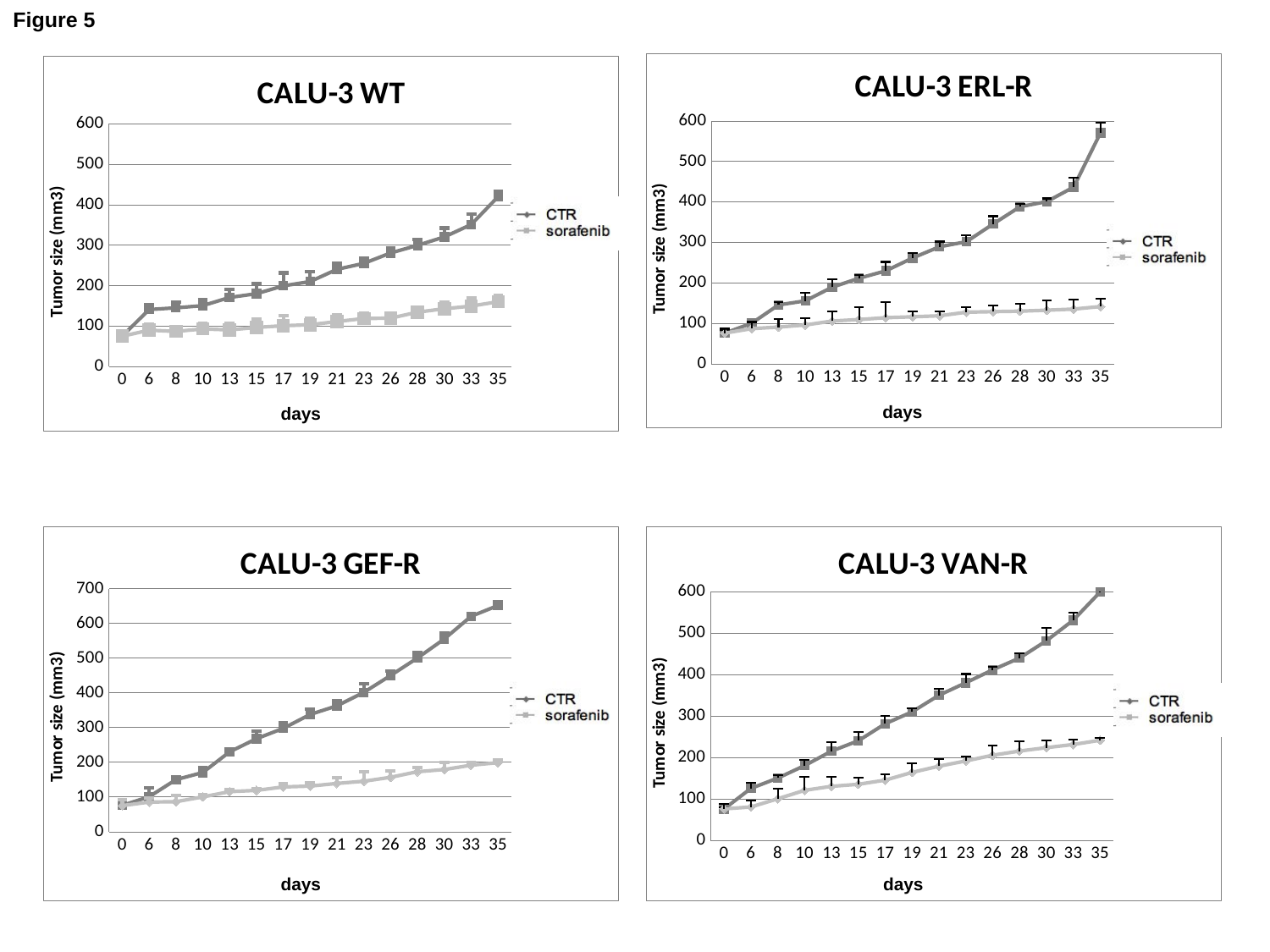

Figure 5
### Chart: CALU-3 ERL-R
| Category | CTR | sorafenib |
|---|---|---|
| 0.0 | 75.720905 | 75.84018 |
| 6.0 | 100.0839775 | 86.86928899999998 |
| 8.0 | 145.314455 | 90.60886899999997 |
| 10.0 | 155.42267 | 95.777994 |
| 13.0 | 189.046845 | 105.507649 |
| 15.0 | 211.15185 | 109.302024 |
| 17.0 | 229.497645 | 113.7877715 |
| 19.0 | 261.2506650000001 | 116.035465 |
| 21.0 | 288.91642 | 118.534975 |
| 23.0 | 301.4130599999998 | 127.431915 |
| 26.0 | 345.2800649999999 | 128.795355 |
| 28.0 | 387.749414 | 129.947545 |
| 30.0 | 400.5077764999999 | 132.415335 |
| 33.0 | 436.2391474999998 | 135.078385 |
| 35.0 | 570.0908525 | 141.369605 |
[unsupported chart]
days
days
### Chart: CALU-3 GEF-R
| Category | CTR | sorafenib |
|---|---|---|
| 0.0 | 75.741705 | 74.98946000000002 |
| 6.0 | 100.62169 | 85.20408 |
| 8.0 | 149.940115 | 86.36478500000001 |
| 10.0 | 170.474135 | 100.006205 |
| 13.0 | 229.7841 | 115.15777 |
| 15.0 | 267.75437 | 118.7576 |
| 17.0 | 297.5086725 | 128.85288 |
| 19.0 | 337.561965 | 131.29142 |
| 21.0 | 362.5716704999999 | 138.912735 |
| 23.0 | 401.1889569999998 | 145.287155 |
| 26.0 | 449.9132039999998 | 156.68432 |
| 28.0 | 500.2191739999998 | 172.6978175 |
| 30.0 | 555.073805 | 178.865691875 |
| 33.0 | 620.31359 | 191.3793375 |
| 35.0 | 651.2735125000003 | 198.72333 |
### Chart: CALU-3 VAN-R
| Category | CTR | sorafenib |
|---|---|---|
| 0.0 | 75.18150250000001 | 75.59334249999998 |
| 6.0 | 125.0890875 | 80.12692999999997 |
| 8.0 | 149.955455 | 99.953815 |
| 10.0 | 180.095045 | 120.284255 |
| 13.0 | 214.9655625 | 130.0903175 |
| 15.0 | 240.15368 | 135.028582 |
| 17.0 | 281.1296345000001 | 145.0280195 |
| 19.0 | 309.9321549999998 | 163.7238395 |
| 21.0 | 350.0238885 | 178.59722075 |
| 23.0 | 380.0912959999998 | 191.2789645 |
| 26.0 | 411.4176299999999 | 205.023234 |
| 28.0 | 439.9615024999998 | 215.12946 |
| 30.0 | 481.507546 | 223.54306 |
| 33.0 | 531.3748765 | 231.097841 |
| 35.0 | 599.4442739999997 | 241.3018595 |
days
days

## Slide 2
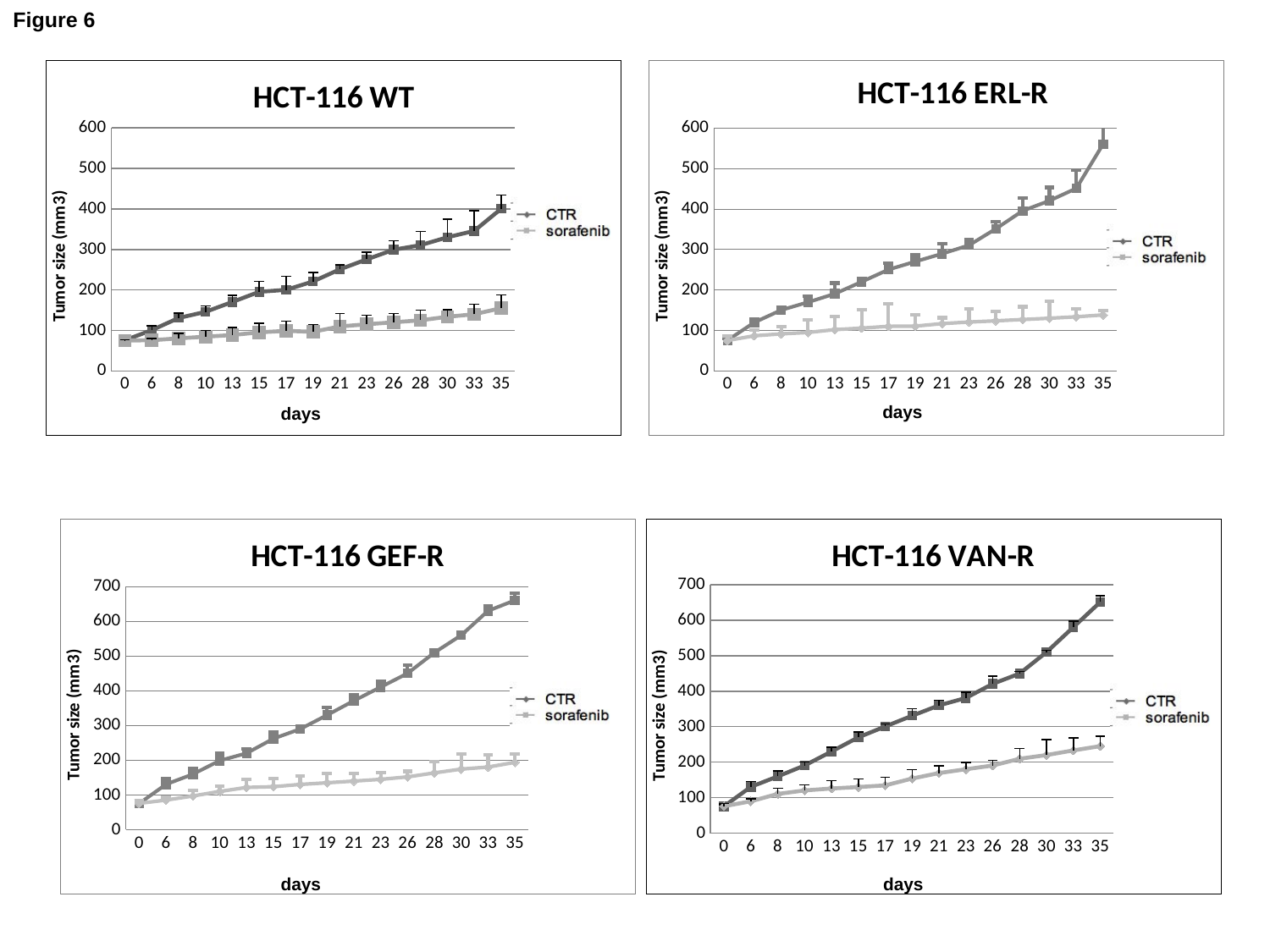

Figure 6
### Chart: HCT-116 WT
| Category | CTR | | |
|---|---|---|---|
| 0.0 | 76.08302 | 75.48567000000001 | None |
| 6.0 | 100.95436 | 76.033126 | None |
| 8.0 | 130.924885 | 80.7422525 | None |
| 10.0 | 146.1288400000001 | 84.861101 | None |
| 13.0 | 170.50839 | 88.925915 | None |
| 15.0 | 195.4749549999999 | 95.5115525 | None |
| 17.0 | 200.311865 | 99.249618 | None |
| 19.0 | 221.0442649999999 | 96.84311 | None |
| 21.0 | 250.92054 | 110.131164 | None |
| 23.0 | 275.442895 | 115.3816105 | None |
| 26.0 | 299.2865472999998 | 120.3898865 | None |
| 28.0 | 310.6210379999998 | 125.2981925 | None |
| 30.0 | 330.0915474999998 | 133.9081055 | None |
| 33.0 | 345.8056836 | 140.13493 | None |
| 35.0 | 400.3502555 | 155.2321615 | None |
### Chart: HCT-116 ERL-R
| Category | CTR | sorafenib |
|---|---|---|
| 0.0 | 75.69464500000002 | 75.204727 |
| 6.0 | 119.794233 | 86.8665915 |
| 8.0 | 150.3383375 | 91.48053849999998 |
| 10.0 | 169.7381855 | 94.95871450000001 |
| 13.0 | 190.4252155 | 101.91311 |
| 15.0 | 220.14044 | 105.671397 |
| 17.0 | 250.27262 | 110.1071465 |
| 19.0 | 270.005125 | 110.6195675 |
| 21.0 | 289.439657 | 116.78069 |
| 23.0 | 310.323052 | 120.8092925 |
| 26.0 | 350.9541164999998 | 123.76559 |
| 28.0 | 395.2870219999999 | 127.00766 |
| 30.0 | 420.3802394999998 | 130.2481245 |
| 33.0 | 450.8485215 | 133.794895 |
| 35.0 | 560.13841 | 138.3831865000001 |
days
days
### Chart: HCT-116 GEF-R
| Category | CTR | sorafenib |
|---|---|---|
| 0.0 | 75.35257599999998 | 75.82239599999996 |
| 6.0 | 130.6474325 | 85.20187 |
| 8.0 | 159.8218635 | 96.961865 |
| 10.0 | 198.919266 | 110.082895 |
| 13.0 | 220.10443 | 121.52569 |
| 15.0 | 262.1775975 | 123.74362 |
| 17.0 | 289.897985 | 130.2370875 |
| 19.0 | 329.9146903 | 134.9051275 |
| 21.0 | 371.4213697999999 | 139.9948875 |
| 23.0 | 410.7575355 | 145.003365 |
| 26.0 | 450.2634369999998 | 151.9677575 |
| 28.0 | 510.006666 | 163.4661925 |
| 30.0 | 560.0926629999998 | 174.4022475 |
| 33.0 | 630.3060074999997 | 180.35017 |
| 35.0 | 660.63478 | 193.83416 |
### Chart: HCT-116 VAN-R
| Category | CTR | sorafenib |
|---|---|---|
| 0.0 | 75.72073599999997 | 75.9977075 |
| 6.0 | 130.5872425 | 89.97446250000002 |
| 8.0 | 159.956485 | 110.7038075 |
| 10.0 | 190.65501 | 120.5531275 |
| 13.0 | 230.00497 | 125.9200215 |
| 15.0 | 270.0780549999998 | 130.097305 |
| 17.0 | 300.508975 | 134.682405 |
| 19.0 | 330.8098950000001 | 153.97252 |
| 21.0 | 360.0438608 | 169.40417 |
| 23.0 | 380.9370825 | 180.139765 |
| 26.0 | 420.6718425 | 190.70714 |
| 28.0 | 449.6826425 | 209.690975 |
| 30.0 | 509.9018014999999 | 220.12029 |
| 33.0 | 580.3813080000001 | 233.098892 |
| 35.0 | 650.9364615000002 | 245.225838 |
days
days
